# ﻿Description of the larva of *Cybisterlewisianus* Sharp, 1873 (Coleoptera, Dytiscidae, Cybistrinae)

**DOI:** 10.3897/zookeys.1197.119508

**Published:** 2024-04-10

**Authors:** Kohei Watanabe, Masakazu Hayashi

**Affiliations:** 1 Ishikawa Insect Museum, Hakusan, Ishikawa, 920–2113, Japan Ishikawa Insect Museum Hakusan Japan; 2 Hoshizaki Green Foundation, Izumo, Shimane, 691–0076, Japan Hoshizaki Green Foundation Izumo Japan

**Keywords:** Chaetotaxy, diving beetle, larval morphology, larval stage, Red List, water beetle

## Abstract

We describe for the first time, the larvae of Cybister (Cybister) lewisianus Sharp, 1873, an endangered species of diving beetle in Japan, emphasizing the chaetotaxy of the cephalic capsule, head appendages, legs, last abdominal segment, and urogomphi. *Cybisterlewisianus* larvae are characterized by a longer third article of antenna 3 than the sum of the first and second articles; rounded apex of parietal setae 1–3; labium seta 8 absent; elongated trochanter seta 4, not multi-branched; rounded apex of abdominal setae 1, 12, and 13 (instar I); narrow lateral projections of the frontoclypeus; pronotum without two dark-brown longitudinal stripes dorsally (instar III); and the base of the thick row of small setae on the inner edge of the mandible angulate and projecting medially (all instars).

## ﻿Introduction

Compared to adults, coleopteran larvae lack morphological information (Hammond et al. 2019). This trend is also present in Dytiscidae, in which information on larval morphology is generally limited (Alarie and Michat 2023). A nomenclature system of Dytiscidae larval chaetotaxy was developed to provide a detailed description and comparison of their larval morphology and to allow for phylogenetic analysis (Alarie and Michat 2023). Particularly, primary sensilla (setae and pores) have been important for the diagnosis and study of phylogenetic relationships among the species (Michat et al. 2017).

The genus *Cybister* Curtis, 1827 includes large diving beetles (adult length: 13–43 mm) and belongs to the subfamily Cybistrinae (Miller and Bergsten 2016). *Cybister* includes 97 species and is distributed in all major biogeographic regions (Miller and Bergsten 2016; Nilsson and Hájek 2023). Seven species are distributed in Japan (Nakajima et al. 2020; Watanabe and Yoshitomi 2022), and six of these species (approximately 86%) are on the national Red List and are threatened with extinction (Ministry of the Environment of Japan 2020).

Knowledge of the larval morphology of *Cybister* is particularly scarce in comparison to that of other diving beetle groups (Miller et al. 2024). Laboratory experiments and field research have gradually unraveled their larval feeding habits in detail (e.g., Ohba 2009a, b; Ohba and Inatani 2012; Yamasaki et al. 2022; Fukuoka et al. 2023). Contrastingly, although the characteristics of larvae of some species have been described (Wilson 1923; Kamiya 1938; Fiori 1949; Morioka 1953; Nakagawa 1954; Fukuda et al. 1959; Watts 1964; Larson et al. 2000; Kamite 2008), information about the larval morphology of only four species can be found, i.e., *Cybisterfimbriolatus* (Say, 1823), *C.lateralimarginalis* (De Geer, 1774), *C.tripunctatus* (Olivier, 1795) and *C.sugillatus* Erichson, 1834 (Michat 2010; Michat et al. 2015, 2017; Miller et al. 2024).

*Cybisterlewisianus* Sharp, 1873 (adult length 21–26 mm) is listed as “Critically Endangered” on the Japanese Red List (Ministry of the Environment of Japan 2015) and is designated a “nationally endangered species of wild fauna and flora” by the Japanese “Conservation of Endangered Species of Wild Fauna and Flora” Act (Ministry of the Environment of Japan 2022). Identifying the larvae of Japanese *Cybister* to species level is challenging due to their similar body shape and length. They have not been described in detail, and morphological information is limited to third-instar larvae (Ichikawa 1984; Kamite 2008; Mitamura et al. 2017). Recently, Inoda et al. (2022) stated that the shape of the larval clypeus could be useful to identify these species. Similarly, Watanabe (2024) suggested color of the antennal segments, ratio of each antennal segment and article, and shape of the mandible are helpful for identification in the field. Herein, we describe in detail the morphometric and chaetotaxic characteristics of *C.lewisianus* larvae for the first time, according to the now generalized system used to describe aquatic Adephaga larvae (Alarie and Michat 2023).

## ﻿Material and methods

All larvae used for description were obtained through rearing following the methods used by Watanabe et al. (2021, 2023). A wild individual (Fig. 4) was captured with official permission of the Wildlife Division, Chubu Regional Environmental Office, Ministry of the Environment of Japan (2303313) and the Ishikawa Prefecture, Japan (4-45), and was released at the site after it was photographed.

The larvae were fixed in boiling water, transferred to 70% ethanol in glass vials with caps and subsequently mounted on slides with 70% ethanol or euparal. The specimens were observed using an optical microscope (Nikon ECLIPSE E400) up to 1000-fold magnification and were sketched using a microscope equipped with a Nikon Y-IDT drawing tube. Figures of the line drawing were prepared using an iPad Pro 11-inch (4^th^ generation) after scanning the sketch. Photographs of the living larvae were captured using a Nikon D500 digital camera equipped with a Nikon AF-S Micro NIKKOR 60 mm f/2.8G ED lens. The examined larvae were deposited in the larval collections of the Ishikawa Insect Museum (Ishikawa, Japan) and the Hoshizaki Green Foundation (Shimane, Japan).

Measurements were made using an optical microscope (Nikon ECLIPSE E400) with a glass slide including a microscale, a stereoscopic microscope (Leica M205C, Planapo 1.0X) with a Leica DFC420 camera, LAS version 3.3.1, and digital vernier calipers (to calculate total length only). The fine structures of the specimens were observed using a JEOL JCM-6000 Neoscope scanning electron microscope (SEM)). Larvae were freeze-dried and coated with ultrathin layers of gold through high-vacuum evaporation. The methods and terms used in this study were abbreviated following Alarie et al. (2011, 2023) and Michat et al. (2015, 2019): A and AN, antenna; AB and LAS, abdominal segment VIII (last abdominal segment); CL, longest claw; CO, coxa; COL, coronal line length; FE, femur; FR, frontoclypeus; FRL, frontoclypeus length; HL, head length; HW, maximum head width; L, leg; LA, labium; LP, labial palpi; MN, mandible; MNL, mandible length; MNW, mandible width; MP, maxillary palpi; MX, maxilla; OCW, occipital foramen width; PA, parietal; PPF, maxillary palpifer; PT, pretarsus; TA, tarsus; TI, tibia; TL, total length; TR, trochanter; U and UR, urogomphus. Primary setae and pores were coded following Alarie et al. (2011) and Alarie and Michat (2023).

## ﻿Results

**Description of larvae of Cybister (Cybister) lewisianus Sharp, 1873**

Figs 1–30, Table 1

**Material source.** The description of larvae of *Cybisterlewisianus* was based on four instar I, three instar II, and three instar III specimens, reared *ex ovo* in the laboratory at the Ishikawa Insect Museum from adults collected in Suzu-shi, Ishikawa Prefecture, with official permission for exhibition and research at the Ishikawa Insect Museum.

**Diagnosis.** Ratio HL/HW = 1.26–1.32 (instar I), 1.30–1.33 (instar II), and 1.32–1.34 (instar III) (Table 1). All instars with base of the thick row of small setae on MN inner edge angulate, projecting medially [not angulate and projecting in *C.brevis* Aubé, 1838, *C.chinensis* Motschulsky, 1854, *C.tripunctatus* (Watanabe 2024)]. Instar I larvae of *C.lewisianus* can be distinguished from those of other *Cybister* species by the following combination of characteristics: EB rounded [spiniform in *C.sugillatus* (Michat et al. 2015)]; first article of A2 pale-yellow [dark-brown in *C.brevis* (Watanabe 2024)]; third article of A3 longer than the sum of first and second articles of A3 [shorter in *C.brevis*, *C.chinensis*, *C.tripunctatus* (Watanabe 2024)]; setae PA1–3 with rounded apex [spiniform in *C.tripunctatus* (Alarie et al. 2011)]; seta FR10 broad [narrow in *C.tripunctatus* (Alarie et al. 2011)]; seta MX11 multi-branched [single in *C.tripunctatus* (Alarie et al. 2011)]; seta LA8 absent [present in *C.sugillatus* and *C.tripunctatus* (Alarie et al. 2011; Michat et al. 2015)]; seta TR4 not multi-branched [multi-branched in *C.tripunctatus* (Alarie et al. 2011)]; setae AB1, AB12, and AB13 with rounded apex [spiniform in *C.tripunctatus* (Alarie et al. 2011)]. In the instar III larva, lateral projections of FR narrow [broad in *C.chinensis*, *C.limbatus* (Fabricius, 1775), *C.rugosus* (MacLeay, 1833) and *C.tripunctatus* (Ichikawa 1984; Kamite 2008; Mitamura et al. 2017; Watanabe and Hayashi 2023)]; pronotum without two dark-brown longitudinal stripes dorsally [with two dark-brown longitudinal stripes in *C.brevis*, *C.sugillatus*, *C.rugosus* (Mitamura et al. 2017; Watanabe and Hayashi 2023)].

**Table 1.** Measurements and ratios for the larvae of *Cybisterlewisianus* Sharp, 1873. *N* = number of specimens examined.

Measure	*C.lewisianus*			Measure	*C.lewisianus*		
	Instar I (*N* = 3)	Instar II (*N* = 3)	Instar III (*N* = 3)		Instar I (*N* = 3)	Instar II (*N* = 3)	Instar III (*N* = 3)
TL (mm)	20.19–20.31	30.37–32.63	50.85–53.36	PPF/MP1	0.36–0.37	0.41–0.42	0.43–0.47
HL (mm)	2.12–2.17	3.27–3.29	4.53–4.82	MP1/MP2	1.52–1.58	1.67–1.81	1.92–1.99
HW (mm)	1.64–1.68	2.46–2.51	3.37–3.61	MP3/MP2	1.28–1.43	1.15–1.22	1.01–1.04
FRL (mm)	0.91–1.07	1.35–1.38	1.74–1.79	MP/LP	2.06–2.25	1.85–2.10	1.92–1.97
OCW (mm)	0.69–0.74	1.14–1.31	1.75–1.98	LP2/LP1	0.76–0.82	0.61–0.66	0.46–0.48
HL/HW	1.26–1.32	1.30–1.33	1.32–1.34	L3 (mm)	5.16–5.43	7.29–7.53	10.23–10.77
HW/OCW	2.28–2.38	1.92–2.17	1.81–1.93	L3/L1	1.20–1.31	1.25–1.30	1.28–1.31
COL/HL	0.51–0.57	0.58–0.59	0.62–0.63	L3/L2	1.12–1.18	1.09–1.16	1.12–1.14
FRL/HL	0.43–0.49	0.41–0.42	0.37–0.38	L3/HW	3.06–3.25	2.96–3.01	2.93–3.03
A/HW	0.95–0.99	0.80–0.84	0.80–0.83	L3 (CO/FE)	1.00–1.02	1.02–1.05	0.98–1.01
A2/A1	0.86–0.94	0.79–0.89	0.75–0.78	L3 (TI/FE)	0.67–0.71	0.66–0.69	0.65–0.67
A3/A1	0.62–0.64	0.53–0.54	0.39–0.40	L3 (TA/FE)	0.74–0.77	0.68–0.69	0.60–0.64
A4/A3	0.17–0.20	0.12–0.14	0.10–0.13	L3 (CL/TA)	0.36–0.37	0.32–0.33	0.26–0.29
A3’/A4	0.81–0.85	0.73–0.96	0.52–0.82	LAS (mm)	4.03–4.26	5.93–6.26	8.30–8.91
MNL/MNW	3.09–3.27	3.16–3.19	3.17–3.30	LAS/HW	2.46–2.53	2.41–2.52	2.43–2.47
MNL/HL	0.49–0.51	0.48–0.50	0.48–0.50	U (mm)	0.02	0.03–0.05	0.04–0.05
A/MP	1.24–1.38	1.37–1.40	1.46–1.51				

**Figures 1–4.**
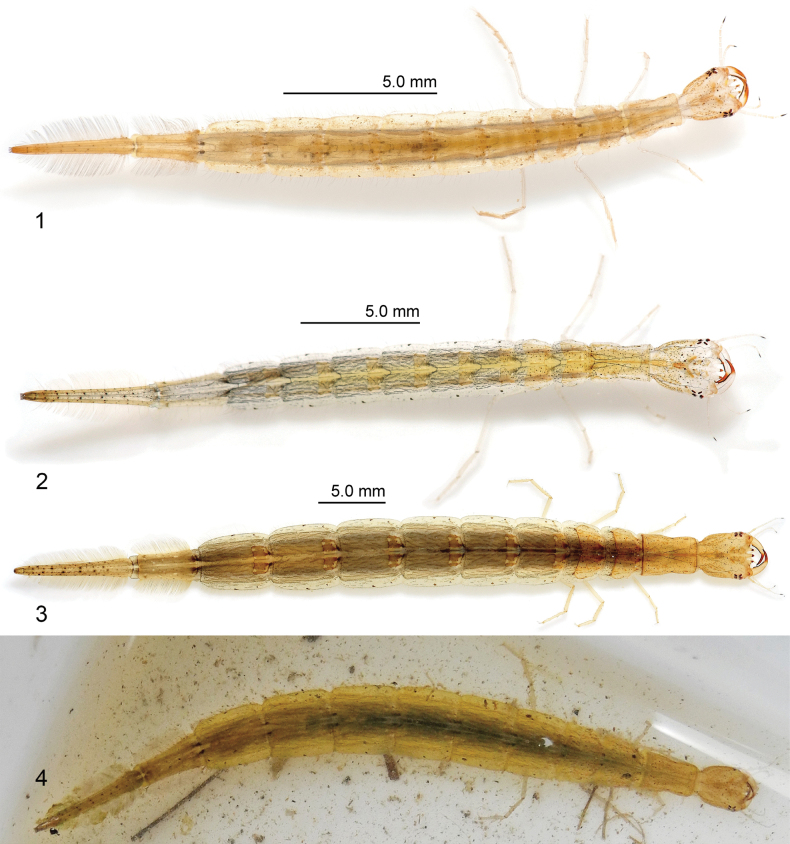
First to third-instar larva of Cybister (Cybister) lewisianus Sharp, 1873 **1** instar I **2** instar II **3, 4** instar III **1–3** captive individuals **4** wild individual.

**Figures 5, 6.**
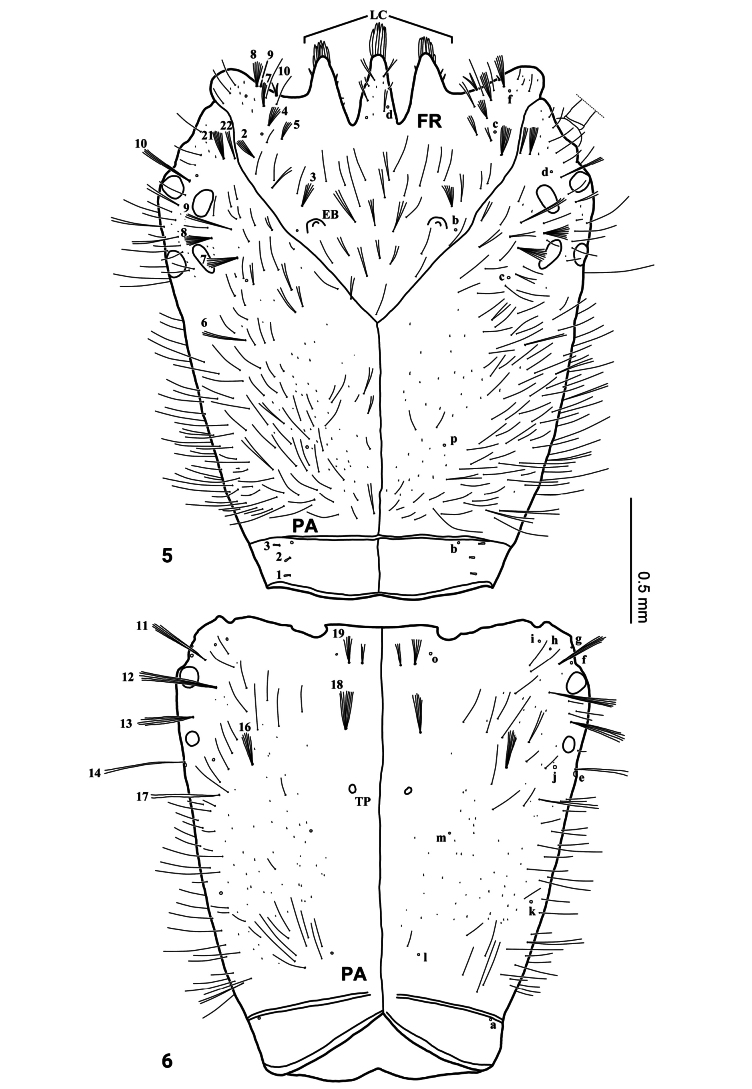
First-instar larva of Cybister (Cybister) lewisianus, cephalic capsule **5** dorsal aspect **6** ventral aspect. EB, egg burster; LC, lamellae clypeales; TP, tentorial pit.

**Figures 7–9.**
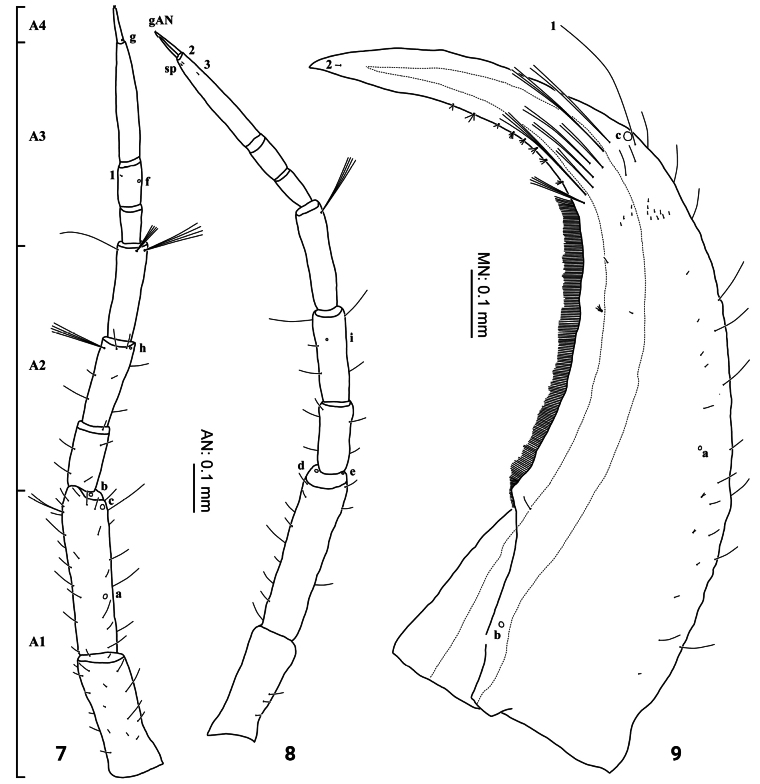
First-instar larva of Cybister (Cybister) lewisianus**7, 8** antenna **9** mandible **7, 9** dorsal aspect **8** ventral aspect. sp, spinula.

**Figures 10–13.**
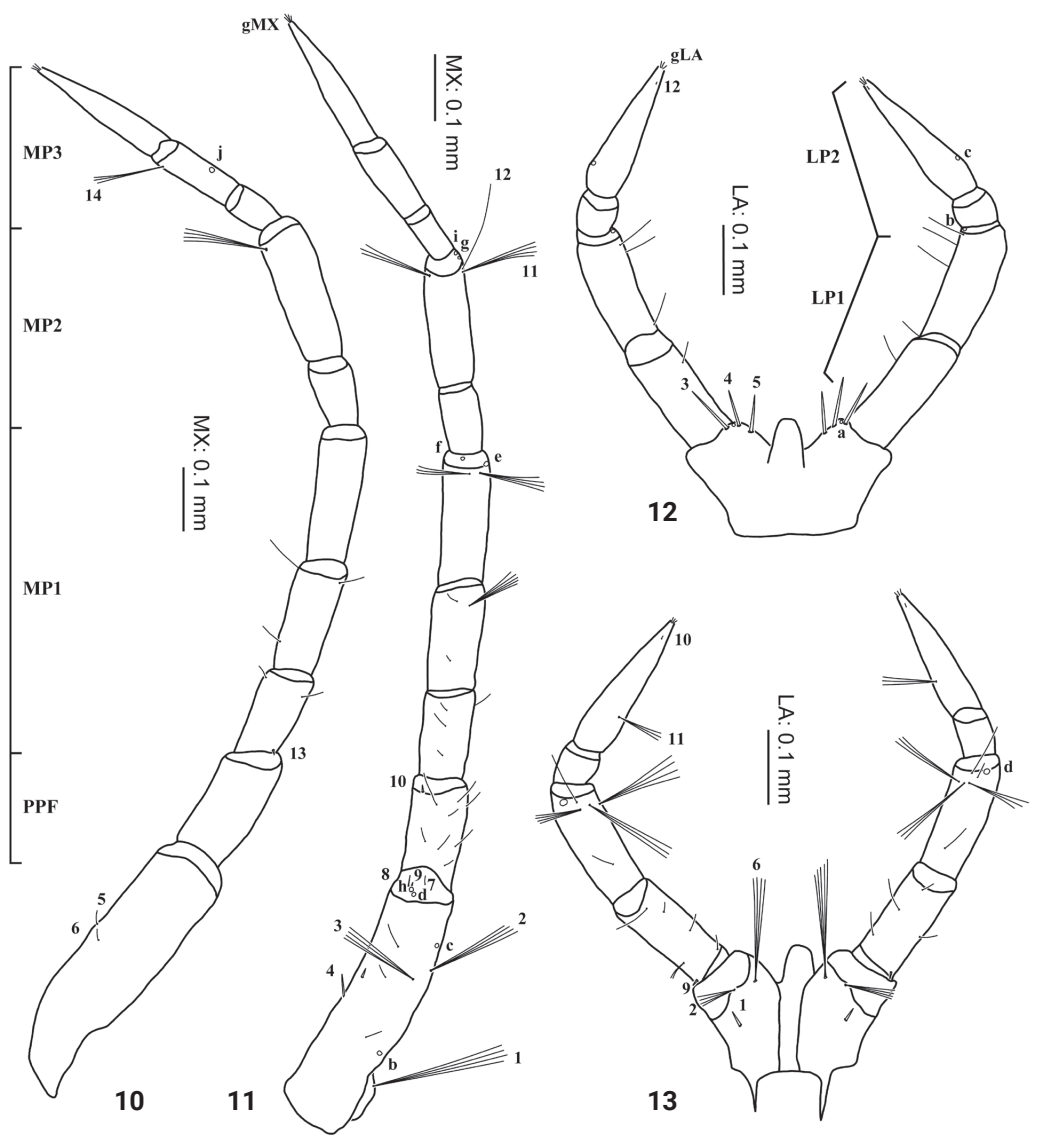
First-instar larva of Cybister (Cybister) lewisianus**10, 11** maxilla **12, 13** labium **10, 12** dorsal aspect **11, 13** ventral aspect.

**Figures 14, 15.**
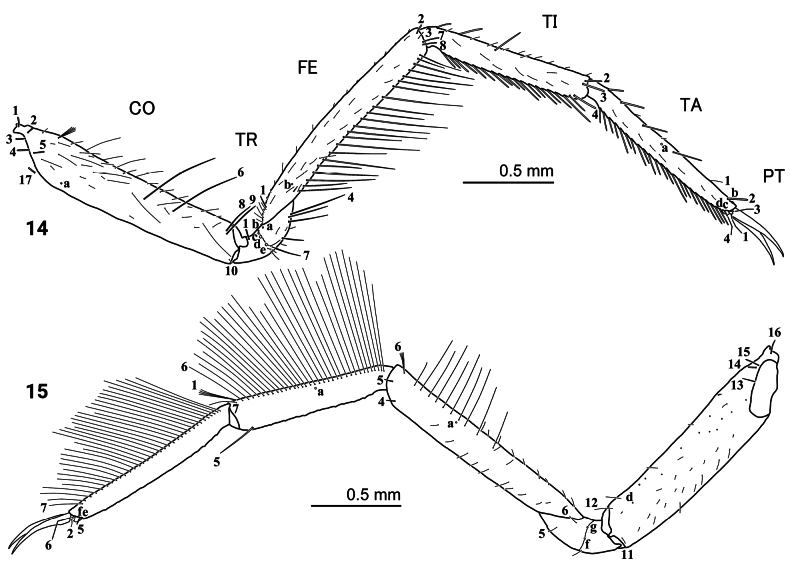
First-instar larva of Cybister (Cybister) lewisianus, metathoracic leg **14** anterior aspect **15** posterior aspect.

**Figures 16–18.**
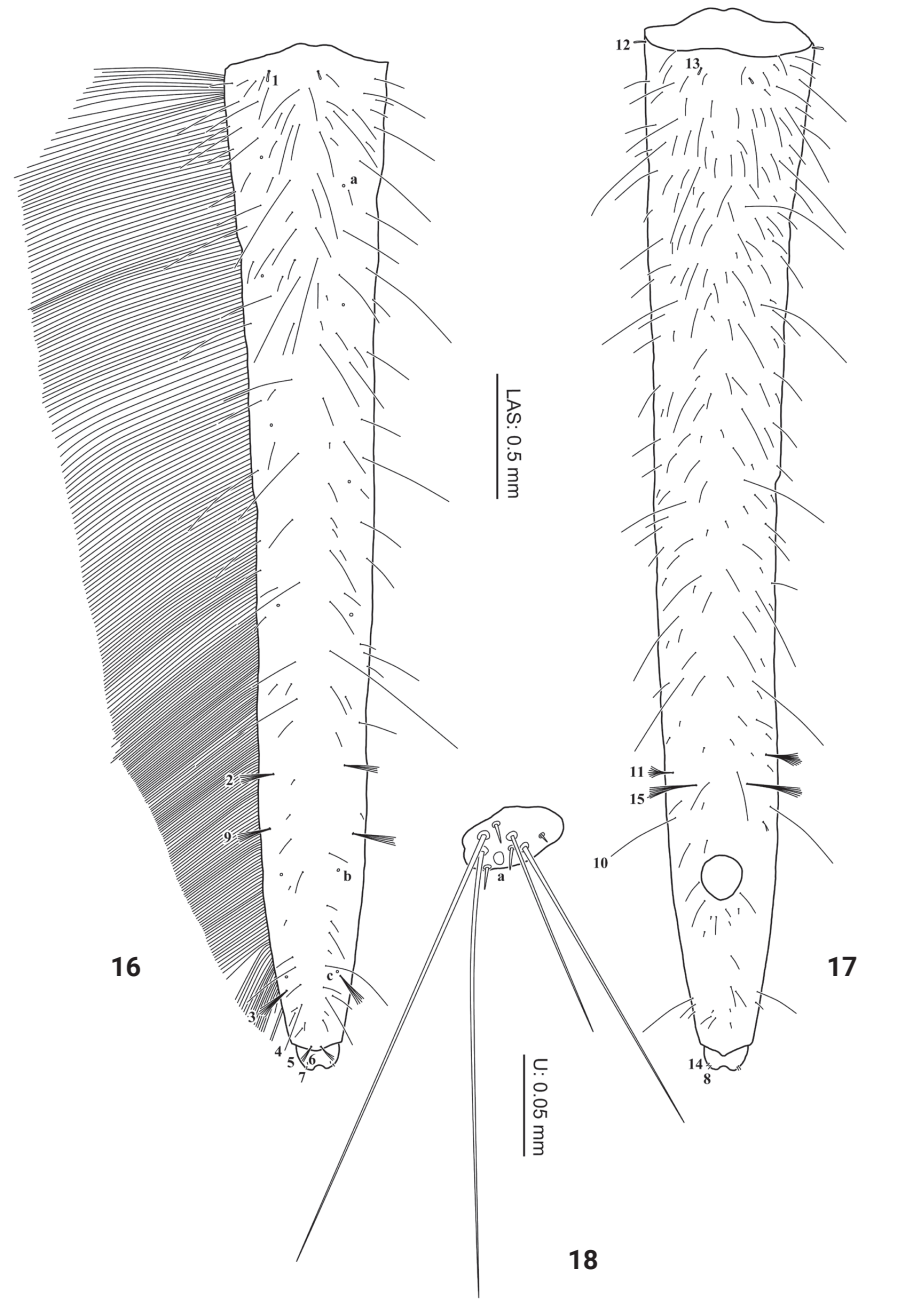
First-instar larva of Cybister (Cybister) lewisianus**16, 17** abdominal segment VIII **18** urogomphus **16** dorsal aspect **17, 18** ventral aspect.

**Figures 19–24.**
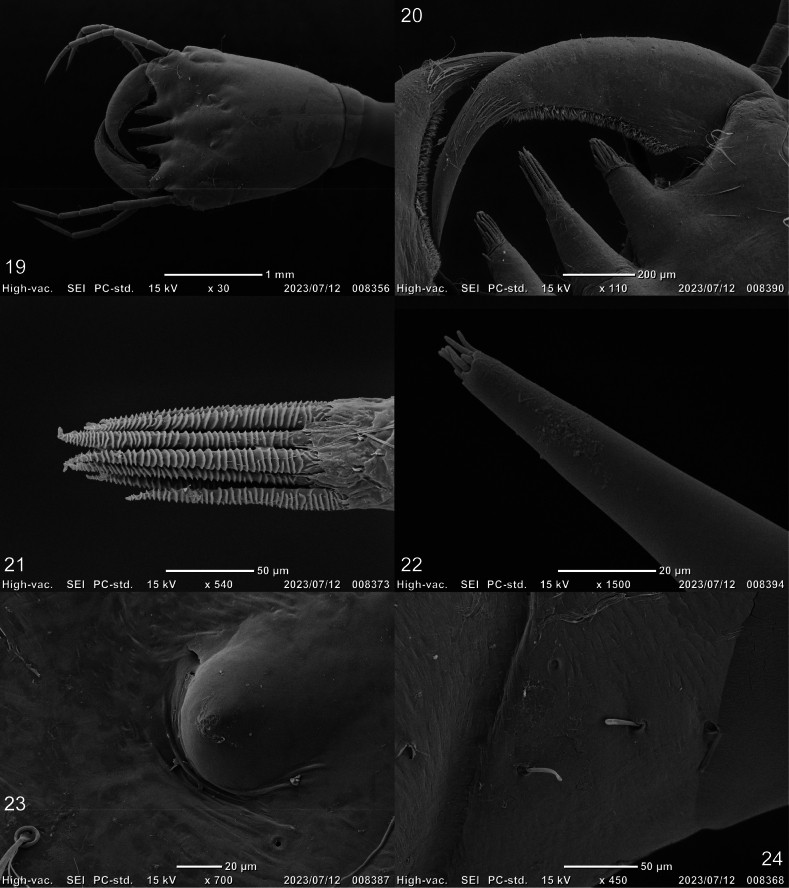
SEM photographs of first-instar larva of Cybister (Cybister) lewisianus, head, dorsal aspect **19** cephalic capsule **20** mandible and projections of lamellae clypeales **21** lamellae clypeales of central projection **22** apex of antenna **23** egg burster **24** setae PA1–3.

**Figures 25–30.**
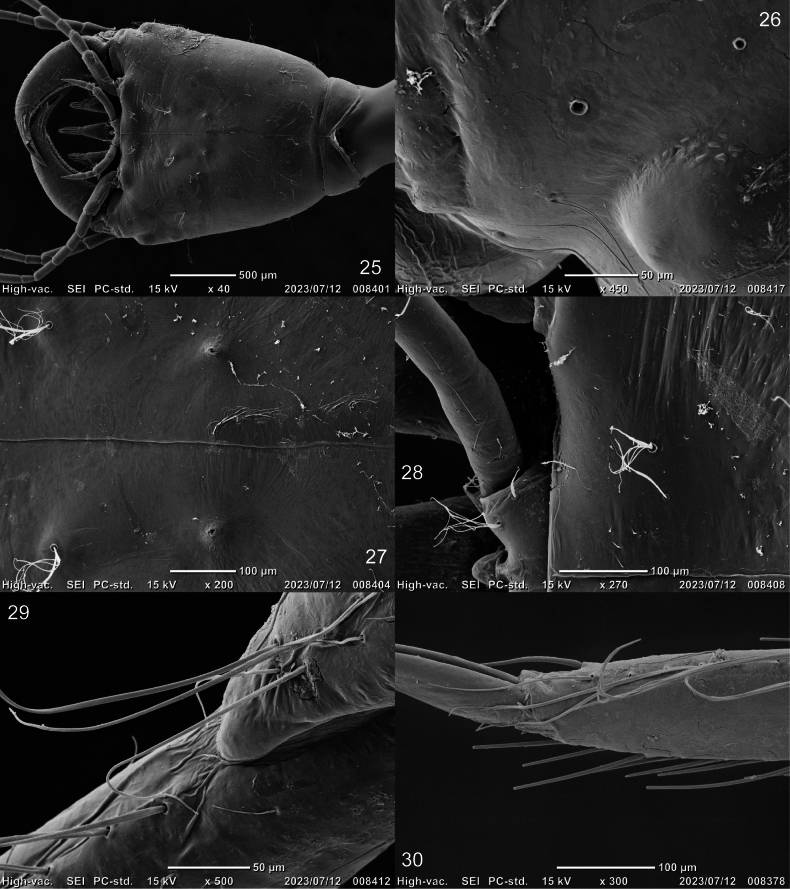
SEM photographs of first-instar larva of Cybister (Cybister) lewisianus**25** cephalic capsule **26** pores PAf–i **27** tentorial pit **28** labium and cephalic capsule **29** trochanter and femur **30** tarsus and pretarsus **25–28** ventral aspect **29** antero-ventral aspect **30** posterior aspect.

**Description, instar I (Figs 1, 5–30). *Color*** (Fig. 1). Head capsule pale-yellow; anterolateral part of frontclypeus, coronal line, and lateral margin light-yellow-brown; several brown small maculae; stemmata dark-brown; antennae pale-yellow except for third article of A3 and A4 dark-brown; mandible pale-yellow except for distal portion light-red-brown and thick row of small setae on the inner margin dark-brown; maxilla pale-yellow except for third article of palpomere 3; labium pale-yellow; thoracic tergites light-yellow-brown except for pale-yellow membranous area, several small brown maculae, slightly larger pair of brown maculae on anterior quarter side of protergite; one pair of brown maculate on each side of membranous region of meso- and metathorax; abdominal tergites I–VI pale-yellow with several small brown maculae; one pair of slightly larger brown maculae on middle of dorsolateral margin of membranous region, five pairs of ventral brown maculae; abdominal tergites VII–VIII light-yellow-brown with several small brown maculae; four pairs of brown maculae on sterna of abdominal tergite VII; legs pale-yellow; urogomphus light-yellow-brown. Color as shown in Fig. 1 and in Watanabe (2024: figs 1B, 2C).

***Body*** (Fig. 1). Elongate, subcylindrical; measurements and body shape ratios are shown in Table 1.

***Head*** (Figs 5–13, 19–28). Cephalic capsule (Figs 5, 6, 19, 25). Flattened, subtriangular, longer than broad; maximum width at level of anterior stemmata, constricted at level of occipital region, lacking temporal spiniform setae; occipital suture present; ecdysial line well marked, COL long; occipital foramen deeply emarginate ventrally (Figs 6, 25); tentorial pits visible slightly above middle of ventral midline (Figs 6, 25, 27); FR subtriangular, anterior margin projected forward, divided into three well developed and about equally long triangular projections, central projection narrow and with same length on both sides, lateral projections slightly broader and with inner length longer than outer length, notches between medial and lateral projections narrow; anterolateral lobes rounded, not projecting beyond lamellae clypeales; EB present, large, rounded (Figs 5, 19, 23), near ecdysial line at level of seta PA9; six stemmata on each side, four dorsal, and two ventral. Antennae (Figs 7, 8, 22). Elongate, slender, almost as long or slightly shorter than HW, composed of four antennomeres; A1 longest, subdivided into two articles, distal article approximately < 1.5 longer than basal article; A2 shorter than A1, subdivided into three articles: first article shortest and third article longest; A3 shorter than A2, subdivided into three articles: first article shortest and third article longest; A3’ shorter than A4, elongate, slender; A4 shortest. Mandible (Fig. 9). Strong, falciform, broadest at base, narrowing to apex, abruptly narrowed toward apex from pore MNc; base of the thick row of small setae on inner edge angulate and projecting medially; mandibular channel present. Maxilla (Figs 10, 11). Premaxillary lobes well developed; cardo well developed, subovate, stipes elongate, slender, subcylindrical; galea absent; PPF elongate, slender, palpomere-like; MP elongate, slender, shorter than antenna, composed of three palpomeres, MP1 longest and MP2 the shortest; MP1 subdivided into three articles, basal article shortest and third article longest, MP2 subdivided into two articles, distal article longer than first article; MP3 subdivided into three articles, first article shortest and third article longest. Labium (Figs 12, 13, 28). Prementum broader than long, not sclerotized ventromedially, anterodorsal margin well projected forward, median process with apex rounded, reaching approximately the level of anterior margin of prementum; LP much shorter than MP, composed of two palpomeres; LP1 longer than LP2; LP1 subdivided into two articles subequal in length; LP2 subdivided into two articles, distal article more than twice longer than basal article.

***Thorax*** (Figs 1, 14, 15, 29, 30). Pro-, meso-, and metanotum convex, length of pronotum twice that of mesonotum, metanotum and mesonotum with subequal length, pronotum, metanotum, and metanotum with subequal width; protergite longer than broad, subrectangular, lateral margins emarginate at about middle, anterior and posterior margins straight; meso- and metatargite small, broader than long, subtrapezoidal, posterior central margin emarginate; sagittal line present on all tergites; sternum of prothorax membranous except for one pair of small subtriangular sclerites, sterna of meso- and metathorax membranous; spiracles absent. Legs (Figs 14, 15, 29, 30). Long, composed of six segments, L1 shortest and L3 longest; CO, FE, TI, and TA subcylindrical, elongate and slender; TR short, divided into two parts by an annulus; PT with two long curved claws.

***Abdomen*** (Figs 1, 16–18). Eight-segmented; segments I–VI subequal in length, mostly membranous with a minute sclerite on anterodorsal region, tergites I–VI without anterior carina, sagittal line present; segments II–III slightly broader than the others; posterior part of sclerites I–VI and anterior part of sclerite VII densely covered with small spinulae; sterna of segments I–VI membranous; segment VII narrower, subtrapezoidal, without anterior carina, sagittal line absent; segments I–VII without spiracles; segment VIII longest and narrowest, sclerotized except anteroventrally and around anus. Urogomphus (Fig. 18). Strongly reduced, slightly broader than long, comprised of one urogomphomere.

***Chaetotaxy*.** Similar to that of the generalized *Cybister* larva (Alarie et al. 2011; Alarie and Michat 2023) with the following remarks: lamellae clypeales drill-like (Figs 20, 21); seta PA1–3 apically rounded (Figs 5, 24); seta FR10 broad, bifid (Fig. 5); seta MX11 multi-branched (Fig. 11); seta LA8 absent (Fig. 12); setae CO1–5, CO14, and CO17 apically rounded (Figs 14, 15); seta TR1 apically rounded (Fig. 14); seta TR4 elongate, not multi-branched (Figs 14, 29); setae FE2, FE7, and FE8 apically rounded (Fig. 14); seta FE3 slenderer than FE2, FE7, and FE8 (Fig. 14); setae TI2–4 apically rounded (Fig. 14); setae TA2–5 apically rounded (Figs 14, 15); seta PT1 apically rounded (Fig. 14); setae AB1, AB12 and AB13 apically rounded (Figs 16, 17); and seta AB4 long (Fig. 16).

**Description, instar II** (Fig. 2). As first-instar larva except as follows:

***Color*** (Fig. 2). Head capsule with small brown maculae more numerous; thoracic tergites light yellow-brown except for pale-yellow membranous region with several gray maculae; abdominal tergites I–VI light-yellow-brown except for pale-yellow membranous region with several gray maculae; abdominal tergites VII–VIII with several small brown maculae. Color as shown in Fig. 2 and in Watanabe (2024: figs 1F, 3B).

***Body*.** Measurements and body shape ratios are shown in Table 1.

***Head*.** Cephalic capsule. EB absent; HW/OCW = 1.92–2.17. Antennae. Shorter than HW; A3/A1 = 0.53–0.54. Maxilla. MP1/MP2 = 1.67–1.81; MP3/MP2 = 1.15–1.22. Labium. LP2/LP1 = 0.61–0.66.

***Abdomen*.** Sclerites I–VII not covered with small spinulae.

***Chaetotaxy*.** Identification of the secondary setae was difficult due to the large number of additional setae.

**Description, instar III (Fig. 3, 4).** As second-instar larva except as follows:

***Color*** (Fig. 3, 4). Head capsule yellow-brown; thoracic tergites yellow-brown except for light-brown membranous region with several gray maculae; anterior and posterior margin of protergite brown; abdominal tergites I–VI yellow-brown except for light-brown membranous region, one light-brown vitta on each side of abdominal tergites; abdominal tergites VII–VIII yellow-brown, tergite VII with several light-yellow-brown maculae. Color as shown in Figs 3, 4 and in Inoda et al. (2022: fig. 1), Watanabe and Hayashi (2023: fig. 4E), and Watanabe (2024: figs 1J, 4B).

***Body*.** Thorax. Spiracles present on mesosternum; measurements and body shape ratios shown in Table 1.

***Head*.** Antennae. A3 shorter than half of A1. Maxilla. MP1 about twice longer than MP2; MP3 almost same length as MP2. Labium. LP2 shorter than half of LP1.

***Abdomen*.** Spiracles present on segments I–VII.

**Remarks.** A study on *Laccophilusyoshitomii* Watanabe & Kamite, 2018 reported that larvae raised in captivity differed in color from those collected in the field (Watanabe and Okada 2023); however, no pronounced differences in color were observed between captive and wild individuals of *Cybisterlewisianus* in the present study (Figs 3, 4).

## ﻿Additional information

### Author ORCIDs

Kohei Watanabe https://orcid.org/0000-0002-8761-232X

Masakazu Hayashi https://orcid.org/0000-0003-3708-9005

### Data availability

All of the data that support the findings of this study are available in the main text.
